# 

**Published:** 1859-06

**Authors:** 


ORIGINAL COMMUNICATIONS.

PAGE.

ART. I. The Causes which affect the Sanitary Condition of Chicago. By DeLaskie Miller, M.D., President of the Chicago Academy of Medical Sciences,......... 325

ART. II. Pregnancy and Extra Uterine Conception. By George Higgins, M.D., of Aurora, Illinois, ........ 334

ART. III. Operation for Ovarian Tumor. By Prof. Meeker, of La Porte, Indiana. Removal of Sequestrum. By Charles Brackett, M.D., Rochester, Indiana....... 337

ART. IV. Sequel and Ultimate Result of a Case of Resection of the Knee Joint. By Daniel Brainard, M.D., Surgeon of the U. S. Marine Hospital at Chicago, 340

ART. V. Three Cases of Inversio Uteri successfully reduced, by Hazard A. Potter, M.D., of Geneva, N. Y. Reported by Chas. M. Hewitt, M.D., with a woodcut, 341

TRANSLATIONS FROM FOREIGN JOURNALS.

Two Memoirs on the Variation of Color in the Venous Blood. By Professor Claude Bernard. Translated by Thomas Bevan, M.D., Chicago,........ 346

EXTRACTS.

Convention of Medical Teachers, assembled at Louisville, May 2d, 1859,........ 356

Twelfth Annual Meeting of American Medical Association. Abstract of Proceedings, 361

BOOK AND PAMPHLET NOTICES.

A Practical Treatise on the Diseases of Infancy and Childhood. By T. H. Tanner, M.D., F.L.S., Licentiate of the Royal College of Physicians; late Physician to the Hospital for Women, etc. etc........ 373

First Annual Report of the Chicago Charitable Eye and Ear Infirmary,........ 379

EDITORIAL.

First Annual Announcement of the Medical Department of the Lind University, at Chicago, Ill., 1859–60,........ 381

MISCELLANEOUS MEDICAL INTELLIGENCE.

Death of Dr. Kirwin, of Ottawa, ........ 385

Letter from F. K. Bailey, M.D., Joliet, ........ 386

Hancock Medical Association, ........ 387

Lithotomy—Excised Knees, ........ 388

